# Cognition in Patients with Sleep-Disordered Breathing: Can Obstructive and Central Apneic Pauses Play a Different Role in Cognitive Impairment?

**DOI:** 10.3390/life12081180

**Published:** 2022-08-02

**Authors:** Patrik Karapin, Pavel Šiarnik, Bianka Suchá, Matúš Jurík, Miroslav Tedla, Michal Poddaný, Katarína Klobučníková, Stanislav Šutovský, Peter Turčáni, Branislav Kollár

**Affiliations:** 1 1st Department of Neurology, Faculty of Medicine, Comenius University, 81499 Bratislava, Slovakia; patrikkarapin@gmail.com (P.K.); sucha50@uniba.sk (B.S.); jurik25@uniba.sk (M.J.); klobucnikova@gmail.com (K.K.); nilusuto@gmail.com (S.Š.); peter.turcani@sm.unb.sk (P.T.); cyklobrano@gmail.com (B.K.); 2 Department of ENT and HNS, Faculty of Medicine, University Hospital Bratislava, Comenius University, 81499 Bratislava, Slovakia; miro.tedla@gmail.com; 3 Institute of Cancer and Genomic Sciences, University of Birmingham, Birmingham B15 2TT, UK; 4 Department of Neurology, General Hospital, 03101 Liptovsky Mikulas, Slovakia; michal.poddany@gmail.com

**Keywords:** sleep-disordered breathing, cognition, cognitive deficit, polysomnography, central apneas, obstructive apneas

## Abstract

Background: There are increasing data linking sleep apnea with cognitive impairment. We aimed to clarify the relationship between sleep-disordered breathing (SDB) and cognition. Detailed attention was assigned to the potential role of central versus obstructive apneic pauses in cognitive impairment. Methods: Patients with suspected SDB were prospectively enrolled, and a complex sleep study was performed that included overnight polysomnography. A revised version of Addenbrooke‘s Cognitive Examination (ACE-R) was used to assess cognition, evaluating overall cognition and individual subdomains. Results: A total number of 101 participants were included in the study. In multivariate binary logistic regression analysis, obstructive apnea index ([OAI], 95% CI: 1.009–1.057, *p* = 0.008) was the only significant contributor to the model predicting attention deficit. The proportion of N1 stage of NREM sleep was the only significant contributor to the model predicting impaired verbal fluency (95% CI: 1.004–1.081, *p* = 0.029). No significant differences in sleep-related indices were observed in the remaining ACE-R subdomains. Conclusion: Except for verbal fluency and attention, we failed to find any significant association of sleep-related indices with the impairment in different cognitive subdomains. Our data suggest that impairment observed in verbal fluency is associated with a higher proportion of shallow NREM sleep, and attention deficit is associated with higher OAI. Obstructive respiratory episodes seem to play a more important role in cognitive impairment when compared to central ones.

## 1. Introduction

Sleep-disordered breathing (SDB) is a treatable disease that is frequent in the adult population [1]. The most common form of SDB is obstructive sleep apnea (OSA), which is characterized by recurrent upper airway obstruction, causing recurrent episodes of apnea or hypopnea during sleep, increased daytime sleepiness, sleep fragmentation, and intermittent hypoxia [2].

There is increased interest in investigating the impact of SDB on overall health and its association with numerous diseases [3,4,5]. Studies are also trying to elucidate the association between OSA and cognitive impairment (CI). Population studies evaluating the relationship between SDB and CI in healthy individuals have mixed results [6,7]. A recent meta-analysis [8] has shown that individuals with SDB have a 26% increased risk of cognitive deficit (risk ratio 1.26; 95% CI 1.05–1.50) when compared to the population without SDB. There are contradictory findings from studies that examined which cognitive domains are mostly impaired in patients with OSA [9]. Attention deficit and executive dysfunction are generally observed in these individuals. On the contrary, memory impairment is less prevalent in patients with OSA [10].

Furthermore, the role of SDB in the deterioration of overall cognition is not precisely recognized. Sleep fragmentation, sleep deprivation, intermittent nocturnal hypoxia, and disruption of sleep architecture are believed to be essential underlying mechanisms responsible for cognitive impairment [10]. Our study aimed to clarify the relationship between SDB and cognitive profile. Detailed attention was paid to the potential role of central versus obstructive apneic pauses in cognitive impairment, which has not been described in detail in previous works.

## 2. Materials and Methods

### 2.1. Participants

We consecutively enrolled patients who were suspected of suffering SDB and were hospitalized in the sleep laboratory of the 1st Department of Neurology, Comenius University, and University Hospital Bratislava (Old Town Hospital, Bratislava, Slovakia). From these patients, participants were selected for our study after meeting the inclusion criteria: (a) age between 18 and 80 years; (b) confirmed diagnosis of SDB by overnight polysomnography (defined as apnea/hypopnea index [AHI] ≥ 5). The exclusion criteria were: (a) presence of a cognitive deficit defined as Mini-Mental State Examination (MMSE) ≤ 24; (b) a known history of psychiatric illness, which may explain the presence of cognitive deficits (depression, psychotic disorders, attention deficit hyperactivity disorder, posttraumatic stress disorder); (c) use of drugs affecting sleep (hypnotics, sedatives, anxiolytics, steroids); (d) history of treatment for sleep disorders. The baseline evaluation of all patients included assessment of clinical and demographic characteristics including sex, age, neck circumference, and body mass index (BMI). Medical records of all patients were reviewed to search for medical conditions (arterial hypertension, diabetes mellitus, atrial fibrillation, stroke, dyslipidemia, and thyroid disease) that could contribute to CI.

All participants agreed to participate in the study. The agreement was confirmed by signing an informed consent prior to enrollment. The study was approved by the Ethics Committee of the Old Town Hospital, University Hospital Bratislava.

### 2.2. Sleep Study

Overnight polysomnography was performed on all participants (Alice 6, Philips-Respironics, Murrysville, PA, USA). The sleep specialist evaluated and scored polysomnography based on standardized criteria [11]. Observed parameters were apnea/hypopnea index (AHI), which was defined as the number of apneic or hypopneas pauses during an hour of sleep; other variables were arousal index ([AI], number of arousals per hour of sleep), desaturation index (number of all oxygen desaturations ≥3% per hour of sleep). We evaluated the obstructive apnea index (OAI), defined as the number of obstructive and mixed apneic pauses per hour of sleep, and the central apnea index (CAI), defined as the number of central apneic pauses per hour of sleep. We also assessed the hypopnea index, defined as the number of hypopneas per hour of sleep and the percentual duration of snoring during sleep. The hypnogram and the percentage of particular sleep stages (REM, NREM phases—N1, N2, and N3) were recorded. The diagnosis of SDB was defined as AHI ≥ 5. The Epworth Sleepiness Scale (ESS) questionnaire was used to assess daytime sleepiness [12], and the Pittsburgh Sleep Quality Index (PSQI) questionnaire was used to subjectively assess sleep quality [13].

### 2.3. Evaluation of Cognition

To assess the cognitive profile, we administered a psychometric examination of cognition in patients prior to polysomnography. An extended screening method was performed—a revised version of Addenbrooke‘s Cognitive Examination (ACE-R) [14]. We evaluated scores of overall cognition and particular cognitive subdomains: orientation/attention, memory, verbal fluency, language, and visuospatial functions. We classified patients according to the presence of an impairment in individual cognitive subdomains. Individual impairment in different subdomains was defined as a subtest score for orientation and attention ≤16; memory subtest score ≤17; verbal fluency subtest score ≤9; language subtest score ≤23; subtest score for visuospatial abilities ≤14 [14].

### 2.4. Statistical Analysis

Statistical analysis was performed using SPSS, version 21 (SPSS Inc, Chicago, IL, USA). Categorical variables were described as number and percentage (%), and continuous variables were described as mean ± standard deviation or median and IQR (interquartile range). To compare values of the continuous variables between two groups, we conducted Student’s *t*-test if variables were normally distributed; otherwise, the Mann–Whitney test was conducted. The Chi-square test was used for categorical variables. *p*-value < 0.05 was considered statistically significant. In the binary logistic regression analysis, 95% confidence intervals were reported to declare the statistical significance and strength of association between deficit in particular cognitive subdomains (dependent variable) and the independent variables (demographic variables: age, gender, body mass index, neck circumference, presence of diseases: arterial hypertension, diabetes mellitus, atrial fibrillation, stroke, dyslipidemia, thyroid disease, and sleep parameters: AHI, AI, desaturation index, the proportion of particular sleep stages, OAI, CAI, hypopnea index, percentual duration of snoring, ESS, and PSQI) was assessed. Variables with a *p* < 0.05 in the bi-variable binary logistic regression analysis were considered for the multivariable analysis.

## 3. Results

### 3.1. Demographic and Cognitive Parameters

After meeting the inclusion criteria, 101 participants were included in the study. Baseline characteristics of the population are shown in Table 1. The decline in ACE-R subtest for orientation and attention was observed in six participants (5.9%). Memory deterioration was present in seven patients (6.9%). Impaired verbal fluency was confirmed in 11 participants (10.9%). Speech deficit was not observed in any individual in the study group. Deterioration of visuospatial functions was observed in 13 patients (12.9%). A CI found in at least one cognitive subdomain was present in 27 individuals (26.7%).

### 3.2. Comparison of Sleep Variables and Cognition

Our primary goal of the study was to compare sleep variables in groups divided according to the presence of CI in different cognitive subdomains. Statistically significant differences between observed sleep parameters were observed in individuals with attention deficit and impaired verbal fluency (detailed results are shown in Table 2). No significant differences in sleep parameters were found in CI subjects in other cognitive subdomains. In participants with attention deficit, we proved that they had statistically higher value of AHI (*p* = 0.035), AI (*p* = 0.039), and desaturation index (*p* = 0.024). In these patients, there were also statistically more frequent obstructive apneic pauses (*p* = 0.025), as well as central apneic pauses (*p* = 0.036). Furthermore, in participants with attention deficit, the ESS value was statistically higher (*p* = 0.012). In patients with impaired verbal fluency, a statistically higher percentage of the N1 NREM phase of sleep was observed (*p* = 0.014). In multivariate binary logistic regression analysis, we found that OAI (95% CI: 1.009–1.057, *p* = 0.008) was the only significant contributor to the model predicting attention deficit. The proportion of the N1 NREM phase was the only significant factor in the model that predicted impaired verbal fluency (95% CI: 1.004–1.081, *p* = 0.029).

## 4. Discussion

The results of our study support the findings of previous works, which emphasize the association between SDB and cognitive impairment. Our results suggest impaired sleep parameters in patients with sleep apnea who also have a CI present in attention and executive functions. Our findings are in agreement with a recent meta-analysis [8], which presents that individuals with OSA had significantly worse executive functions than the population without OSA. On the other hand, there was no association with impaired global cognition or memory decline.

The findings of our work support previous findings about the high prevalence of CI in individuals with SDB. We also found that alterations in cognitive functions were associated with the presence of sleep apnea, the severity of nocturnal desaturations, sleep fragmentation, and increased daytime sleepiness [10,15]. Our findings suggest that recurrent apneic pauses with subsequent intermittent brain hypoxia and sleep fragmentation could affect cognitive impairment. The consequences of these processes can cause structural and cerebrovascular damage to the brain, which could ultimately explain cognitive impairment in patients with SDB [16]. However, the design of our study does not allow us to elucidate the causal role of sleep apnea in the process of cognitive impairment.

Altered sleep architecture and sleep disruption could be important underlying mechanisms that link SDB with cognitive decline. Our results suggest that in individuals with SDB, alterations in cognition were associated with a higher proportion of shallow NREM sleep. It could indicate a deterioration in restorative sleep function and possible subsequent negative effects on cognitive function. This hypothesis is supported by the findings of previous studies, where the reduction in REM sleep and increased percentage of N1 NREM phase correlated with attention deficit and executive dysfunction [17,18]. In our study, the proportion of the N1 NREM phase was the only significant factor in the model predicting impaired verbal fluency.

A possible causal link could be increased amyloid production in disruption of NREM sleep. Sleep duration is disrupted and shortened during physiological aging and in the development of Alzheimer’s disease (AD). Furthermore, a significant decrease in slow-wave sleep is observed, which correlates with the N3 stage of NREM sleep [19]. These changes are more prominent in patients with mild CI and individuals with AD [20,21]. A bidirectional relationship is suspected between the reduction of deep NREM sleep phases and pathomorphological changes in AD [22]. Animal studies support this hypothesis. Experimental increase in cortical amyloid β (Aβ) deposition causes NREM sleep disruption [23]. On the other hand, experimental NREM sleep reduction and prolonged wakefulness result in increased Aβ production [24]. In contrast, NREM sleep promotes the clearance of extracellular Aβ, which accumulates during wakefulness [25].

The strength of our work is the assessment of the impact of obstructive versus central apneic pauses on cognitive profile. Most studies deal with OSA and its possible impact on cognition. We are aware of only a few studies that took central sleep apnea into account. In a recent study, the authors assessed the prevalence of different types of SDB in elderly patients. In this study, the authors found that OSA was associated with lower general cognition, while CSA was only associated with executive dysfunction [26]. We found that individuals with attention deficit had statistically more frequent obstructive and central apneas. This finding could lead to the assumption that both obstructive and central apneas are linked to attention deficit. This hypothesis could be explained by the fact that obstructive and central apnea overlap in some clinical consequences [27]. On the other hand, after evaluating multivariate binary logistic regression analysis, only the OAI and not CAI was associated with attention deficit. This could support the superior importance of obstructive apneas in the pathogenesis of attention deficit. The exact underlying mechanisms need to be elucidated by future prospective studies. Increased cerebrovascular morbidity in OSA patients is well-known and could play a role. On the other hand, the causal role of CSA in the pathogenesis of the cerebrovascular disease remains controversial [28].

The conclusions of our study could be affected by several limitations. The main limitation of our work is a relatively small study population. We are aware that multiple factors could impact the results in performed cognitive tests, including premorbid intellect or achieved education that were not studied in detail in our study. Another factor that may have interfered with our results is the presence of undiagnosed depression, but we tried to influence this modifying factor by the exclusion of participants who had been diagnosed with depression or other significant psychiatric illnesses that could interfere with cognition.

## 5. Conclusions

Our study confirmed the high frequency of cognitive deficit in subjects with SDB. Except for verbal fluency and attention, we failed to find any significant association of sleep-related indices with the deficit in other cognition subdomains. Our data suggest that alteration of verbal fluency was associated with a higher proportion of N1 NREM sleep. Attention deficit was associated with higher OAI. Obstructive respiratory episodes seem to play a more important role in cognitive deficit when compared to central ones.

## Figures and Tables

**Table 1:** Baseline characteristics.

Demographic Variables		Sleep Parameters		Cognitive Parameters	
**Number of participants** (**%**)	101 (100%)	**AHI** (***n*/hour**)	31.8; 36.7 (5.8–157.2)	**MMSE**	28; 2 (25–30)
**Men’s representation** (**%**)	70 (69.3%)	**Arousal index** (***n*/hour**)	18; 25.3 (1.4–89)	**ACE-R**	94; 7 (81–100)
**Age** (**years**)	55.08 ± 11.51	**Desaturation index** (***n*/hour**)	29.3; 39.2 (1.2–149.6)	**Orientation-attention**	18; 1 (16–18)
**BMI** (**kg/m^2^**)	33.86 ± 6.67	**REM** (**%**)	8.5; 10.4 (0–30.4)	**Memory**	23; 3 (13–26)
**Neck circumference** (**cm**)	42.37 ± 4.15	**N1** (**%**)	34.1; 22.9 (1.9–74.6)	**Verbal fluency**	12; 3 (7–14)
**Arterial hypertension** (**%**)	69 (68.3%)	**N2** (**%**)	27.8; 18.7 (6.3–82.7)	**Language**	26; 0 (24–26)
***Diabetes mellitus*** (**%**)	21 (20.8%)	**N3** (**%**)	23.5; 21.9 (0–58.1)	**Visuospatial functions**	16; 1 (12–16)
**Atrial fibrillation** (**%**)	9 (8.9%)	**OAI** (***n*/hour**)	5.72; 18.24 (0–132.28)		
**Stroke** (**%**)	5 (5.0%)	**CAI** (***n*/hour**)	5.44; 12.87 (0–92.75)		
**Dyslipidemia** (**%**)	31 (30.7%)	**Hypopnea index** (***n*/hour**)	15.41; 18.17 (1.26–65.86)		
**Thyroid disease** (**%**)	14 (13.9%)	**Snoring** (**%**)	3.9; 10.65 (0–42.4)		
		**ESS**	7; 8 (0–24)		
		**PSQI**	5; 4 (0–17)		

BMI—body mass index; ESS—Epworth Sleepiness Scale; PSQI—Pittsburgh Sleep Quality Index; AHI—apnea/hypopnea index; REM—percentage of REM phase during the whole sleep; N1—percentage of N1 NREM phase during the whole sleep; N2—percentage of N2 NREM phase during the whole sleep; N3—percentage of N3 NREM phase during the whole sleep; OAI—obstructive apnea index; CAI—central apnea index; MMSE—Mini-Mental State Examination; ACE-R—Addenbrooke‘s Cognitive Examination-Revised; Snoring—percentual duration of snoring during sleep.

**Table 2:** Baseline characteristics in populations with and without a deficit in the particular cognitive subdomain.

Variables	Attention			Verbal Fluency		
	Without Deficit	With Deficit	*p*-Value	Without Deficit	With Deficit	*p*-Value
**Number of participants** (**%**)	95 (94.1%)	6 (5.9%)		90 (89.1%)	11 (10.9%)	
**Men’s representation** (**%**)	65 (68.4%)	5 (83.3%)	0.442	65 (72.2%)	5 (45.5%)	0.069
**Age** (**years**)	55.41 ± 11.17	49.83 ± 16.38	0.252	54.82 ± 11.30	57.18 ± 13.51	0.524
**BMI** (**kg/m^2^**)	33.57 ± 6.59	38.58 ± 6.81	0.074	33.91 ± 6.59	33.48 ± 7.65	0.842
**Neck circumference** (**cm**)	42.21 ± 4.18	44.83 ± 2.99	0.134	42.32 ± 3.93	42.73 ± 5.93	0.762
**AHI** (***n*/hour**)	31.8; 37.6 (6.7–153.2)	64.9; 67.9 (21.0–140.3)	0.035 *	34.9; 44.9 (6.7–153.2)	31.2; 16.3 (15.2–72.9)	0.819
**Arousal index** (***n*/hod.**)	16.8; 24.7 (1.4–89)	38; 27.2 (16.8–71.8)	0.039 *	18.9; 26.4 (1.4–89)	16; 7.7 (10.8–38.9)	0.586
**Desaturation index** (***n*/hour**)	27.4; 36.6 (1.2–149.6)	66.1; 60.4 (17.5–120.1)	0.024 *	31.3; 41.1 (1.2–149.6)	25.1; 13.9 (7.5–70.1)	0.42
**REM** (**%**)	8.4; 10.3 (0–25.7)	11.65; 15.4 (0–30.4)	0.551	8.55; 9.8 (0–30.4)	2.9; 11.9 (0–15.8)	0.138
**N1** (**%**)	34; 23.1 (1.9–74.6)	37.35; 36.2 (8.2–66.4)	0.537	31.35; 20.7 (1.9–74.6)	45.4; 19 (23–72.3)	0.014 *
**N2** (**%**)	28; 19.5 (6.3–82.7)	24.5; 11.1 (18.8–36.4)	0.413	28.15; 19.8 (6.3–82.7)	25.9; 8.1 (8.3–37.9)	0.206
**N3** (**%**)	23.5; 23.2 (0–58.1)	22.9; 9.6 (14.8–36.2)	0.886	24; 23.5 (0–58.1)	21.8; 16.5 (11–39.2)	0.731
**OAI** (***n*/hour**)	5.33; 13.76 (0–119.18)	31.6; 66.18 (0.77–132.28)	0.025 *	5.97; 19.22 (0–132.28)	4.66; 7.31 (0–20.45)	0.38
**CAI** (***n*/hour**)	4.79; 12.12 (0–92.75)	23.25; 31.53 (4.26–37.57)	0.036 *	5.75; 13.54 (0–92.75)	3.35; 7.26 (0.72–37.57)	0.922
**Hypopnea index** (***n*/hour**)	14.2; 40.6 (0–169.4)	19.45; 47.7 (1.2–133.5)	0.886	14.75; 39.25 (0–163.8)	16.3; 63.1 (0–169.4)	0.407
**Snoring** (**%**)	3.60; 10.70 (0–42.40)	4.65; 17.05 (0.3–34.9)	0.556	3.75; 10.58 (0–40.9)	4.0; 15.0 (0–42.4)	0.883
**ESS**	7; 8 (0–20)	13; 10 (7–24)	0.012 *	7; 8 (0–24)	6; 8 (1–16)	0.922
**PSQI**	5; 4 (0–14)	6; 9 (2–17)	0.319	5; 4 (0–17)	8; 5 (4–14)	0.079

* *p* < 0.05. BMI—body mass index; ESS—Epworth Sleepiness Scale; PSQI—Pittsburgh Sleep Quality Index; AHI—apnea/hypopnea index; REM—percentage of REM phase during the whole sleep; N1—percentage of N1 NREM phase during the whole sleep; N2—percentage of N2 NREM phase during the whole sleep; N3—percentage of N3 NREM phase during the whole sleep; OAI—obstructive apnea index; CAI—central apnea index; snoring—percentual duration of snoring during sleep.

## Data Availability

The data presented in this study are available on request from the corresponding author.
